# The Effects of Vitamin C Supplementation on Corneal Endothelial Damage in Hard Cataract Phacoemulsification: an Oxidative Stress Study on Aqueous Humour and Corneal Endothelial Cell Characteristics, A Randomized, Double-Blinded, Clinical Trial

**DOI:** 10.12688/f1000research.170846.1

**Published:** 2025-10-15

**Authors:** Syska Widyawati, Ratna Sitompul, Septelia I Wanandi, Melva Louisa, Aria Kekalih, Rina LDR Nora

**Affiliations:** 1 Ophthalmology, Faculty of Medicine, University of Indonesia, Jakarta, Jakarta, 10430, Indonesia; 2 Biochemistry and Molecular Biology, Faculty of Medicine, University of Indonesia, Jakarta, Jakarta, 10430, Indonesia; 3 Pharmacology and Therapeutics, Faculty of Medicine, University of Indonesia, Jakarta, Jakarta, 10430, Indonesia; 4 Community Medicine, Faculty of Medicine, University of Indonesia, Jakarta, Jakarta, 10430, Indonesia

**Keywords:** ascorbic acid, phacoemulsification, oxidative stress, hard nucleus cataract.

## Abstract

**Background:**

Phacoemulsification is one of the most frequent surgeries in the world. However, prolonged use of phacoemulsification machines produces reactive oxygen species which will damage corneal endothelial cells. Ascorbic acid has an antioxidant capacity to neutralize oxidative stress in the anterior chamber. This study will investigate the protective effect of ascorbic acid on corneal endothelial cells in patients with hard nuclear cataracts.

**Methods:**

This study is a double-blinded randomized controlled trial. Samples will be divided into three groups, and 500 mg of vitamin C, tid, or placebo will be received for seven weeks. Clinical characteristics, ascorbic acid, malondialdehyde, and total antioxidant capacity of patients in serum and aqueous humor will be measured before and after intervention and phacoemulsification.

**Conclusions:**

Data from this study will reveal the protective effect of oral vitamin C supplementation on the corneal endothelial cells in patients with hard nucleus cataracts.

This trial has been registered at ClinicalTrials.gov (identifier: NCT06781970; registered on 17 January 2025). The trial record is available at: https://clinicaltrials.gov/ct2/show/NCT06781970

## Introduction

Cataract is the most probable cause of blindness in Indonesia. Universally, WHO estimated that 51% of blindness in the world is caused by cataracts and there are 20 million people who are blind from cataracts. In 2020, the prevalence of blindness in Indonesia is 1.4% with 81.2%, or three million, being from cataracts. ^1^ This number progressively increases as aging is the risk factor for cataracts and is positively correlated to cataract density. With 10% of the total population in Indonesia being of geriatric population in 2021, it is predicted there will be more than 50 million elders in Indonesia in 2045 suffering from cataracts. ^1^

Phacoemulsification is the most used cataract extraction method, with a high success rate of 80.1% compared to extracapsular cataract extraction (ECCE) and intracapsular cataract extraction (ICCE). ^1^ Phacoemulsification utilizes ultrasonic waves, up to 20,000 Hz, to break apart and emulsify the lens. Mechanic vibration of the probe causes bubbles through the acoustic cavitation phenomenon in the aqueous humor. These bubbles, if popped, will dissociate water molecules and produce reactive free radicals. ^1^ Phacoemulsification is safer and more effective than other cataract extraction methods. However, higher power and longer duration of ultrasonic waves from the phacoemulsification probe may cause postoperative complications through the increased number of free radicals. ^2^

Free radicals bind with antioxidants in tissues. The imbalance between the number of free radicals and antioxidants in the tissue causes oxidative stress. ^3^ On a molecular level, stress oxidative can be measured through various markers, one of which is malondialdehyde (MDA). MDA is the end-product of phospholipid oxidation, in which the phospholipid bond is one of the main targets of reactive oxygen species.

Corneal endothelial cells (CEC) have little to no regeneration capacity, hence CEC damage is irreversible. Naturally, corneal endothelial cells diminish 0.3-0.6% yearly. ^4^ This phenomenon is aggravated by increased levels of oxidative stress. Phacoemulsification causes a 40% increase in intraocular free radicals if not followed by sufficient antioxidants. Patients with hard nucleus cataracts undergo a longer duration of phacoemulsification and higher phaco power, hence there is a four times higher risk of endothelial decompensation. Permanent endothelial decompensation usually observed 4-6 weeks postoperatively, is a condition called bullous keratopathy. ^5^ The definitive management of bullous keratopathy is a corneal transplant. However, the ratio of donor availability and demand is 1:70. ^6^ There is a need for an alternative solution.

Total antioxidant capacity (TAC) portrays the capacity of a tissue to neutralize oxidants. As much as 73% of TAC in intraocular tissue is ascorbic acid (AA). ^7^ The amount of AA and TAC in the aqueous humor is found to be an independent protective factor towards endothelial decompensation. An increase of 1 mM TAC in aqueous humour causes 50 times lowered risk of endothelial decompensation. Protective features of AA towards oxidative stress in the cornea have been proven in vitro and in animal studies. In a rabbit corneal endothelial culture, topical AA is found to lower cell apoptosis. ^8, 9^ Using AA in irrigation solution in phacoemulsification for dogs is found to lower endothelial cell density, hexagonality, and variation coefficient. ^10^ On mild-to-moderate degree cataract, 500 mg of AA qid. lowers endothelial cell density difference to 30% compared to control. ^11^

To our knowledge, there is yet a clinical study to investigate the protective effect of oral AA in hard nucleus cataracts. This manuscript describes the protocol for a clinical trial to analyze the protective effects of oral ascorbic acid on the corneal endothelial layer undergoing oxidative stress due to phacoemulsification in patients with hard cataracts.

## Protocol

### Study design

This is a double-blinded, randomized controlled trial investigating the protective effect of ascorbic acid supplementation on corneal endothelial cells undergoing phacoemulsification. The study will follow the SPIRIT (Standard Protocol Items: Recommendations for Interventional Trials) guidelines.

### Study setting

This study is going to be conducted in the Faculty of Medicine, University of Indonesia with samples from a regional hospital in Cianjur, West Java and Serang, Banten, Indonesia.

### Aims

The primary aim of this study is to analyze the protective effect of oral ascorbic acid supplementation on corneal endothelial cells which undergo oxidative stress due to phacoemulsification in patients with hard nucleus cataracts. This study will investigate clinical parameter changes in all three intervention groups, including endothelial cell density (ECD) loss, central corneal thickness, visual acuity, and cells in the anterior chamber.

This study will investigate differences in MDA levels in aqueous humor, prior to and after phacoemulsification, and compare between groups with vitamin C supplementation and those without. We will also investigate differences in AA, TAC, and MDA levels in serum and aqueous humor at the start of the study and after seven days of supplementation and compare them between the three groups. Furthermore, we will explore the correlation between aqueous humor and serum AA, TAC, and MDA levels.

### Participants

The target population of this study is patients with hard nucleus cataracts who undergo phacoemulsification. Patients are eligible to be included in this study with the following inclusion criteria: aged above 60 years, have senile cataract in one or both eyes with Lens Opacities Classification System (LOCS) III nuclear opacity grade 4-6 and nuclear color grade 4-6, willing to consume prescription and participate in the study follow up for seven weeks starting from recruitment, and signed the informed consent. Written informed consent was obtained from all participants prior to enrollment in the study.

The exclusion criteria of this study are as follows: patients with vitamin C allergy or sensitivity, undergo intraoperative complications of postoperative infections, have corneal endothelium disorder, history of intraocular surgery, glaucoma, trauma, or other intraocular inflammations, have diabetes mellitus and kidney disorders, or consumes vitamins as daily supplements.

Samples will be dropped out during the study if they did not participate in the study follow-up or consume less than 80% of the prescribed medications.

### Screening

We screen patients who sign up for a mass cataract surgery event in a regional hospital in Cianjur, West Java, Indonesia. Patients will be asked for information regarding personal identification, history of systemic and ocular diseases, and allergies. They then will undergo ophthalmology examination including uncorrected visual acuity (UCVA) and best corrected visual acuity (BCVA), intraocular pressure using non-contact tonometry (NCT), biomicroscopy slit lamp, and indirect fundoscopy to evaluate anterior and posterior chambers. Lens opacity will then be measured using LOCS III criteria. We will also examine ECD, coefficient of variety (COV), and hexagonality of endothelial cell layers using a specular microscope (REM 4000, Rodenstock, Germany), central corneal thickness (CCT) using Anterior Segment Optical Coherence Tomography (ASOCT), and biometry. Blood samples will be then drawn from patients to measure serum AA, MDA, and TAC.

### Intervention

Patients will be divided into three groups, each with 40 samples. The first group (n=40) will receive oral vitamin C, tid for one week before surgery to four weeks after surgery. The second group (n=40) will receive oral vitamin C, tid for one week before surgery and a placebo after surgery. The last group (n=40) will receive a placebo one week before surgery to four weeks after surgery. Every phacoemulsification is operated by equally experienced surgeons and identical phacoemulsification machines and consumables.

### Post-intervention evaluation

Patients will be asked to come a week after screening. Firstly, they asked the side effects of the supplementation, including gastrointestinal disorders (nausea, diarrhea, epigastric pain). Second blood samples will be then drawn to measure post-intervention serum AA, MDA, and TAC.

During surgery, after the initial incision, 0.3 mL of aqueous humor will be taken using a 1 cc syringe with a 30 G cannula. Phacoemulsification will then be conducted using standard operating procedure to intraocular lens implantation. Another 0.3 mL of aqueous humor will then be taken three minutes after removing the viscoelastic as a post-phacoemulsification sample. All patients will receive topical medications: levofloxacin 5 mg/mL every three hours for one week, prednisolone acetate 10 mg/mL every three hours for 1 week, and tapered down in 4-6 weeks.

Every patient will be evaluated one day, one week, four weeks, and six weeks post-phacoemulsification to measure study parameters and signs of complications, i.e. inflammation or infection. Patients who experience side effects or complications, including toxic anterior segment syndrome (TASS) or endophthalmitis during the duration of follow-up will be exempted from the study and managed according to the standard operational procedure of the Department of Ophthalmology, Faculty of Medicine, University of Indonesia.

### Participant compensation

Participants receive IDR100,000 at the end of the last follow-up.

### Statistical analysis

Data from this study will be recorded and analyzed by IBM SPSS 20.0. Sample personal identification, including demographic and clinical characteristics, will be reported in a descriptive analysis. Data distribution of numeric variables will be measured using the Saphiro-Wilk test and will be represented in mean and standard deviation if the distribution is normal, and median and range if the distribution is not normal. Nominal data will be reported in frequency and percentage. We will use Pearson’s chi-squared test for categorical data. Independent T-test or Mann-Whitney test will be used for comparing numeric data, and paired T-test or Wilcoxon test will be used for paired numeric data. Pearson’s test will be used to test the correlation between data in aqueous humor and serum.

**Dissemination**

The findings from this study will be disseminated through presentations at national and international conferences and submitted for publication in peer-reviewed journals. Trial outcomes will also be reported in the trial registry ( ClinicalTrials.gov). Authorship will follow ICMJE recommendations. The investigators intend to publish the findings regardless of the direction or significance of the results.

The results of this study will provide data on the protective effect of vitamin C supplementation for corneal endothelial cells in patients with hard nucleus cataracts undergoing phacoemulsification. This will initiate further investigation for the alternative management for permanent endothelial decompensation or bullous keratopathy.

**Study status**

At the time of manuscript submission (September 2025), the study is in the data processing phase. Trial registration has been completed ( ClinicalTrials.gov Identifier: NCT06781970).

**Ethical considerations**

This study was approved by the the Faculty of Medicine, University of Indonesia – Cipto Mangunkusumo Hospital Ethics Committee with reference number KET-1252/UN2.F1/ETIK/PPM.00.02/2024 (protocol number: 24-08-1198). All participants provided written informed consent prior to participation.

## Data Availability

No data are associated with this article as it describes a study protocol. Data generated from the study will be made openly available upon completion. No underlying data are associated with this article as it describes a study protocol. The completed SPIRIT 2013 checklist for this study is publicly available in Zenodo: SPIRIT checklist for “The Effects of Vitamin C Supplementation on Corneal Endothelial Damage in Hard Cataract Phacoemulsification: an Oxidative Stress Study on Aqueous Humour and Corneal Endothelial Cell Characteristics, A Randomized, Double-Blinded, Clinical Trial”. Zenodo. DOI: https://doi.org/10.5281/zenodo.17239356. ^12^ License: CC0 1.0 Universal.
