# Antioxidant Support for the Corneal Endothelium: Evidence from Vitamin C Supplementation in Hard Cataract Phacoemulsification

**DOI:** 10.12688/f1000research.170846.3

**Published:** 2026-04-24

**Authors:** Syska Widyawati, Ratna Sitompul, Septelia I Wanandi, Melva Louisa, Aria Kekalih, Rina LDR Nora

**Affiliations:** 1 Ophthalmology, Faculty of Medicine, University of Indonesia, Jakarta, Jakarta, 10430, Indonesia; 2 Biochemistry and Molecular Biology, Faculty of Medicine, University of Indonesia, Jakarta, Jakarta, 10430, Indonesia; 3 Pharmacology and Therapeutics, Faculty of Medicine, University of Indonesia, Jakarta, Jakarta, 10430, Indonesia; 4 Community Medicine, Faculty of Medicine, University of Indonesia, Jakarta, Jakarta, 10430, Indonesia

**Keywords:** ascorbic acid, phacoemulsification, oxidative stress, hard nucleus cataract.

## Abstract

**Background:**

Phacoemulsification is one of the most frequent surgeries in the world. However, prolonged use of phacoemulsification machines produces reactive oxygen species which will damage corneal endothelial cells. Ascorbic acid has an antioxidant capacity to neutralize oxidative stress in the anterior chamber. This study will investigate the protective effect of ascorbic acid on corneal endothelial cells in patients with hard nuclear cataracts.

**Methods:**

This study is a double-blinded randomized controlled trial. Samples will be divided into three groups, and 500 mg vitamin C three times daily (1500 mg/day), or placebo will be received for seven weeks. Clinical characteristics, ascorbic acid, malondialdehyde, and total antioxidant capacity of patients in serum and aqueous humor will be measured before and after intervention and phacoemulsification.

**Conclusions:**

Data from this study will reveal the protective effect of oral vitamin C supplementation on the corneal endothelial cells in patients with hard nucleus cataracts.

This trial has been registered at ClinicalTrials.gov (identifier: NCT06781970; registered on 17 January 2025). The trial record is available at: https://clinicaltrials.gov/ct2/show/NCT06781970

## Introduction

Cataract is the most probable cause of blindness in Indonesia. Universally, WHO estimated that 51% of blindness in the world is caused by cataracts and there are 20 million people who are blind from cataracts. In 2020, the prevalence of blindness in Indonesia is 1.4% with 81.2%, or three million, being from cataracts. This number progressively increases as aging is the risk factor for cataracts and is positively correlated to cataract density. With 10% of the total population in Indonesia being of geriatric population in 2021, it is predicted there will be more than 50 million elders in Indonesia in 2045 suffering from cataracts. ^1^^,^^2^

Phacoemulsification is the most used cataract extraction method, with a high success rate of 80.1% compared to extracapsular cataract extraction (ECCE) and intracapsular cataract extraction (ICCE). ^3^ Phacoemulsification utilizes ultrasonic waves, up to 20,000 Hz, to break apart and emulsify the lens. Mechanic vibration of the probe causes bubbles through the acoustic cavitation phenomenon in the aqueous humor. These bubbles, if popped, will dissociate water molecules and produce reactive free radicals. ^3^ Phacoemulsification is safer and more effective than other cataract extraction methods. However, higher power and longer duration of ultrasonic waves from the phacoemulsification probe may cause postoperative complications through the increased number of free radicals. ^4^

Free radicals bind with antioxidants in tissues. The imbalance between the number of free radicals and antioxidants in the tissue causes oxidative stress. ^5^ On a molecular level, oxidative stress can be measured through various markers, one of which is malondialdehyde (MDA). MDA is the end-product of phospholipid oxidation, in which the phospholipid bond is one of the main targets of reactive oxygen species.

Corneal endothelial cells (CEC) have little to no regeneration capacity, hence CEC damage is irreversible. Naturally, corneal endothelial cells diminish 0.3-0.6% yearly. ^6^ This phenomenon is aggravated by increased levels of oxidative stress. Phacoemulsification causes a 40% increase in intraocular free radicals if not followed by sufficient antioxidants. Patients with hard nucleus cataracts undergo a longer duration of phacoemulsification and higher phaco power, hence there is a four times higher risk of endothelial decompensation. Permanent endothelial decompensation is typically observed 4–6 weeks postoperatively, is a condition called bullous keratopathy. ^7^ The definitive management of bullous keratopathy is a corneal transplant. However, the ratio of donor availability and demand is 1:70. ^8^ There is a need for an alternative solution.

Total antioxidant capacity (TAC) portrays the capacity of a tissue to neutralize oxidants. As much as 73% of TAC in intraocular tissue is ascorbic acid (AA). ^9^ The amount of AA and TAC in the aqueous humor is found to be an independent protective factor towards endothelial decompensation. An increase of 1 mM TAC in aqueous humour causes 50 times lowered risk of endothelial decompensation. Protective features of AA towards oxidative stress in the cornea have been proven in vitro and in animal studies. In a rabbit corneal endothelial culture, topical AA is found to lower cell apoptosis. ^10, 11^ Using AA in irrigation solution in phacoemulsification for dogs is found to lower endothelial cell density, hexagonality, and variation coefficient. ^12^ On mild-to-moderate degree cataract, 500 mg of AA qid. lowers endothelial cell density difference to 30% compared to control. ^13^

Vitamin C has been widely studied for its potential to mitigate corneal endothelial damage caused by oxidative stress during phacoemulsification. Previous studies have demonstrated protective effects of vitamin C in mild to moderate cataracts using oral supplementation, as well as beneficial effects of topical administration in experimental animal models. ^10, 11, 13^ Oral vitamin C may represent a practical antioxidant due to its wide availability and lower risk of adverse effects compared with other antioxidant strategies such as intravenous vitamin C and intracameral antioxidants. Regarding these findings, we hypothesized that oral vitamin C supplementation increases ascorbic acid levels and overall antioxidant capacity in the aqueous humor and reduces oxidative stress–related endothelial damage during phacoemulsification. However, to date, no clinical studies have specifically evaluated the protective role of oral vitamin c supplementation in patients with hard cataracts undergoing phacoemulsification. On the other hand, aqueous humor will also be replaced by irrigation fluid during phacoemulsification, which may reduce the intraocular antioxidant content. Therefore, oral vitamin C is given postoperatively to restore AA levels in the aqueous humor. Thus, it is expected that the antioxidant effect of AA can suppress postoperative corneal endothelial autophagy and apoptosis, which can cause severe damage to the endothelial layer. Considering that aqueous humor sampling cannot be performed during the postoperative recovery period, it is important to determine whether the levels of MDA, TCA, and AA in the aqueous humor are the same as or correlate with blood levels, as no studies have yet examined these three biomolecular parameters simultaneously in both compartments.

## Protocol

## Methods

### Study design

This is a double-blinded, randomized controlled trial investigating the protective effect of ascorbic acid supplementation on corneal endothelial cells undergoing phacoemulsification. The study will follow the SPIRIT (Standard Protocol Items: Recommendations for Interventional Trials) guidelines.

### Study setting

This study is going to be conducted in the Faculty of Medicine, University of Indonesia with samples from a regional hospital in Cianjur, West Java and Serang, Banten, Indonesia.

### Aims

The aim of this study is to analyze the protective effect of oral ascorbic acid supplementation on corneal endothelial cells which undergo oxidative stress due to phacoemulsification in patients with hard nucleus cataracts. The primary outcome will investigate mean change and percentage in endothelial cell density (ECD) loss prior to and after phacoemulsification, assessed at postoperative follow-up period (day 1, week 1, week 4, and week 6) of patients with hard nucleus cataracts.

The secondary endpoint will investigate mean change in other clinical parameters such as central corneal thickness (CCT), visual acuity, and cells in the anterior chamber during the postoperative follow-up period. This study will investigate differences in MDA levels in aqueous humor, prior to and after phacoemulsification, and compare between groups with vitamin C supplementation and those without. We will also investigate differences in AA, TAC, and MDA levels in serum and aqueous humor at the start of the study and after seven days of supplementation and compare them between the three groups. Furthermore, we will explore the correlation between aqueous humor and serum AA, TAC, and MDA levels.

### Sample size calculation

A total sampling method was used. The sample size was determined based on the primary outcome of postoperative corneal endothelial cell density (ECD) loss, referring to a previous study by Chee et al. ^14^ The minimal sample size calculated for this study was 36 subjects for each group (95% confidence interval, power 80%, β = 0.20, α = 0.05, SD = 201 ​​cell/mm ^2^, expected difference = 132 ​​cell/mm ^2^, subjects should have at least 132 ​​cell/mm ^2^ difference of ECD loss after phacoemulsification in the intervention group). With a 10% additional for anticipating loss to follow-up subjects, the minimal sample size needed was 40 for each group.

### Participants

The target population of this study is patients with hard nucleus cataracts who undergo phacoemulsification. Patients are eligible to be included in this study with the following inclusion criteria: aged above 60 years, have senile cataract in one or both eyes with Lens Opacities Classification System (LOCS) III nuclear opacity grade 4-6 and nuclear color grade 4-6, willing to consume prescription and participate in the study follow up for seven weeks starting from recruitment, and signed the informed consent. Written informed consent was obtained from all participants prior to enrollment in the study.

The exclusion criteria of this study are as follows: patients with vitamin C allergy or sensitivity, undergo intraoperative complications of postoperative infections, have corneal endothelium disorder, history of intraocular surgery, glaucoma, trauma, or other intraocular inflammations, have diabetes mellitus and kidney disorders, or consumes vitamins as daily supplements.

Samples will be dropped out during the study if they did not participate in the study follow-up or consume less than 80% of the prescribed medications.

### Screening

We screen patients who sign up for a mass cataract surgery event in a regional hospital in Cianjur, West Java, Indonesia. Patients will be asked for information regarding personal identification, history of systemic and ocular diseases, and allergies. They then will undergo ophthalmology examination including uncorrected visual acuity (UCVA) and best corrected visual acuity (BCVA), intraocular pressure using non-contact tonometry (NCT), biomicroscopy slit lamp, and indirect fundoscopy to evaluate anterior and posterior chambers. Lens opacity will then be measured using LOCS III criteria. We will also examine ECD, coefficient of variety (COV), and hexagonality of endothelial cell layers using a specular microscope (REM 4000, Rodenstock, Germany), central corneal thickness (CCT) using Anterior Segment Optical Coherence Tomography (ASOCT), and biometry. Blood samples will be then drawn from patients to measure serum AA, MDA, and TAC.

### Randomization, blinding and allocation concealment

**Figure 1.**
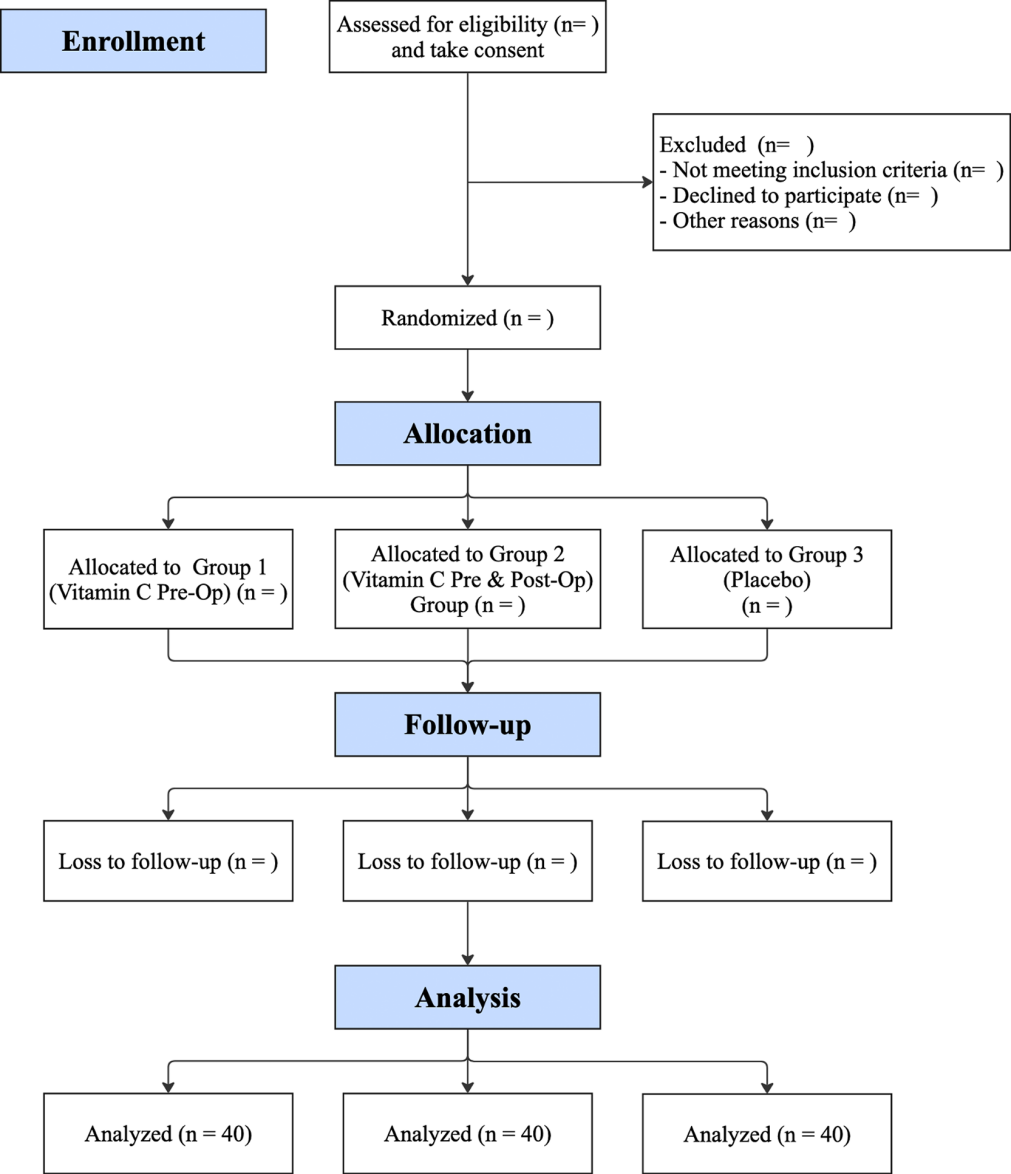
Planned CONSORT flow diagram of participants screening, randomization, follow-up, and analysis.

The subjects will be allocated into three groups: Group 1 will receive oral vitamin C 500 mg, three times daily pre- and postoperatively, Group 2 will receive it only preoperatively, and Group 3 will receive a placebo. Participants will be randomly assigned to the study groups using a computer-generated block randomization sequence with a block size of four. The randomization sequence will be prepared by an independent researcher who will not be involved in patient recruitment or outcome assessment. Allocation concealment will be ensured using sequentially numbered, sealed, opaque envelopes. The study will be conducted in a double-blinded manner. Operating surgeons, examiners, laboratory analysts, and study participants will be blinded to group allocation throughout the study period. Participants will be followed up for seven weeks, and data will be analyzed after the minimum required number of participants has been reached. Figure 1 shows the planned CONSORT flow diagram of participants screening, randomization, follow-up, and analysis.

### Intervention

Patients will be divided into three groups, each with 40 samples. The first group (n=40) will receive 500 mg oral vitamin C three times daily (1500 mg/day) for one week before surgery to four weeks after surgery. The second group (n=40) will receive 500 mg oral vitamin C three times daily (1500 mg/day) for one week before surgery and a placebo after surgery. The last group (n=40) will receive a placebo one week before surgery to four weeks after surgery. Oral vitamin C 1500 mg/day was chosen based on pharmacokinetic evidence showing that this dosage can achieve stable plasma concentrations and approach a steady-state level during short-term administration. ^15^

To minimize potential bias related to surgical variability, all phacoemulsification procedures will be performed using standardized surgical techniques. The surgeries will be conducted by experienced cataract surgeons using the same phacoemulsification machine and standardized operative settings. Key intraoperative parameters, including ultrasound power, irrigation parameters, and the type of ophthalmic viscosurgical device (OVD), will be standardized across all procedures.

### Post-intervention evaluation

Patients will be asked to come a week after screening. Firstly, they asked the side effects of the supplementation, including gastrointestinal disorders (nausea, diarrhea, epigastric pain). Second blood samples will be then drawn to measure post-intervention serum AA, MDA, and TAC.

Sampling for oxidative stress in the aqueous humour will be performed twice, pre- and post-phacoemulsification. During surgery, after the initial incision, 0.2 mL of aqueous humor will be taken using a 1 cc syringe with a 30 G cannula. Phacoemulsification will then be conducted using standard operating procedure to intraocular lens implantation. Another 0.2 mL of aqueous humor will then be taken three minutes after removing the viscoelastic as a post-phacoemulsification sample. This sampling volume is within the range commonly used in aqueous humor biomarker studies and is considered safe under surgical conditions. Because the sampling will be performed through the surgical incision and the anterior chamber will subsequently be maintained with a balanced salt solution, anterior chamber stability will not be affected. ^16, 17^ The pre-phacoemulsification aqueous humour samples will be analysed for ascorbic acid (AA), total antioxidant capacity (TAC), and malondialdehyde (MDA), while the post-phacoemulsification samples will be analysed for MDA levels only. Intraoperative parameters, including effective phacoemulsification time (EPT) and total surgical duration, will be recorded for each procedure. All patients will receive topical medications: levofloxacin 5 mg/mL every three hours for one week, prednisolone acetate 10 mg/mL every three hours for 1 week, and tapered down in 4-6 weeks.

Every patient will be evaluated one day, one week, four weeks, and six weeks post-phacoemulsification to measure study parameters and signs of complications, i.e. inflammation or infection. Patients who experience side effects or complications, including toxic anterior segment syndrome (TASS) or endophthalmitis during the duration of follow-up will be exempted from the study and managed according to the standard operational procedure of the Department of Ophthalmology, Faculty of Medicine, University of Indonesia.

### Laboratory analysis

The examination of blood and aqueous humor samples will be conducted at the Pharmacology Laboratory, Faculty of Medicine, Universitas Indonesia (FMUI). Blood samples will be collected from all study participants who will have provided written informed consent. The collection, storage, and transportation procedures will be carried out under strict control of time and temperature to maintain sample stability. All participants will be gathered at the same time, and blood samples will be collected sequentially within a short period so that the entire process, from the first to the last sample, will be completed within one hour.

Immediately after collection, blood samples will be temporarily stored in a cooler box and will be transported from Sayang General Hospital, Cianjur, to the Pharmacology Laboratory, FMUI, under police escort from the Cianjur Police Department. The total transport time will be less than two hours. Centrifugation will be performed within six hours after collection at a speed of 1,000G for 15 minutes to obtain plasma, will be then stored at −80°C until further analysis.

Participants will be randomly assigned into three groups using computer-generated randomization: one placebo group and two intervention groups receiving oral vitamin C supplementation at predetermined doses. Each participant will be provided with a logbook containing the follow-up schedule, adverse effect monitoring, and contact information in case of any complaints. A second blood sample will be collected after seven days of supplementation for the analysis of ascorbic acid (AA), total antioxidant capacity (TAC), and malondialdehyde (MDA).

Aqueous humor samples will be collected twice, each of 0.2 mL: first, immediately after the incision (pre-phacoemulsification), and second, three minutes after viscoelastic removal following intraocular lens implantation (post-phacoemulsification). Both aqueous humor samples will be stored in a cool box containing dry ice at −78°C and will subsequently be transferred to long-term storage at −80°C in the Pharmacology Laboratory, FMUI, before analysis.

Principles of Analysis:

1. Measurement of Ascorbic Acid (AA) Levels
Ascorbic acid levels will be determined using an Enzyme-Linked Immunosorbent Assay (ELISA) Kit for Vitamin C (Cat. No. CEA913Ge). The principle of this assay is based on a competitive inhibition enzyme immunoassay method.
2. Measurement of Total Antioxidant Capacity (TAC)
Total antioxidant capacity will be measured using the QuantiChrom™ Antioxidant Assay Kit (DTAC-100, BioAssay Systems). This assay is based on the cupric ion reducing antioxidant capacity (CUPRAC) method.
3. Measurement of Malondialdehyde (MDA) Levels
  • MDA in Aqueous Humor:
Malondialdehyde levels in aqueous humor will be determined using a Malondialdehyde ELISA Kit (ab287797, Abcam). The principle of this assay is based on an indirect sandwich enzyme-linked immunosorbent assay (ELISA) for the quantitative detection of MDA in serum, plasma, or other biological fluids.
  • MDA in Blood Samples:
Malondialdehyde levels in blood samples will be measured using a spectrophotometric method based on the reaction of MDA with thiobarbituric acid (TBA) to form a pink-colored complex that can be quantified spectrophotometrically.

### Participant compensation

Participants receive IDR100,000 at the end of the last follow-up.

### Statistical analysis

Data from this study will be recorded and analyzed by IBM SPSS 20.0. Sample personal identification, including demographic and clinical characteristics, will be reported in a descriptive analysis. Data distribution of numerical variables will be assessed using the Shapiro–Wilk test with normal distribution will be presented as mean (standard deviation [SD]); non-normal distribution will be reported as median (interquartile range [IQR]). Nominal data will be reported in frequency and percentage. We will use Pearson’s chi-squared test for categorical data. For comparisons among the three groups, one-way ANOVA will be used for normally distributed data, while the Kruskal–Wallis test will be used for non-normally distributed data. Significant group differences will be followed by post-hoc pairwise comparisons using Bonferroni correction for ANOVA or Dunn’s test following the Kruskal–Wallis analysis to account for multiple testing. Pearson’s test will be used to test the correlation between data in aqueous humor and serum. A p-value of less than 0.05 (p < 0.05) will be considered statistically significant.

Effective phacoemulsification time (EPT) and total surgical duration will be included as covariates in the statistical analysis to account for their potential influence on postoperative endothelial outcomes. Multivariable analysis will also be conducted to control for potential confounding variables, including smoking status, occupational exposure, ultraviolet (UV) light exposure, and dietary intake of vitamin C. These variables will be collected through structured questionnaires during the screening.

**Dissemination**

The findings from this study will be disseminated through presentations at national and international conferences and submitted for publication in peer-reviewed journals. Trial outcomes will also be reported in the trial registry ( ClinicalTrials.gov). Authorship will follow ICMJE recommendations. The investigators intend to publish the findings regardless of the direction or significance of the results.

The results of this study will provide data on the protective effect of vitamin C supplementation for corneal endothelial cells in patients with hard nucleus cataracts undergoing phacoemulsification. This will initiate further investigation for the alternative management for permanent endothelial decompensation or bullous keratopathy.

**Study status**

At the time of manuscript submission (September 2025), the study is in the data processing phase. Trial registration has been completed ( ClinicalTrials.gov Identifier: NCT06781970).

**Ethical considerations**

This study was approved by the Institutional Review Board of the Faculty of Medicine, Universitas Indonesia – Cipto Mangunkusumo Hospital Ethics Committee with Approval number: KET-1252/UN2.F1/ETIK/PPM.00.02/2024 on 30 ^th^ August 2024. All participants provided written informed consent prior to participation.

## Data Availability

No data are associated with this article as it describes a study protocol. Data generated from the study will be made openly available upon completion. No underlying data are associated with this article as it describes a study protocol. The completed SPIRIT 2013 checklist for this study is publicly available in Zenodo: SPIRIT checklist for “The Effects of Vitamin C Supplementation on Corneal Endothelial Damage in Hard Cataract Phacoemulsification: an Oxidative Stress Study on Aqueous Humour and Corneal Endothelial Cell Characteristics, A Randomized, Double-Blinded, Clinical Trial”. Zenodo. DOI: https://doi.org/10.5281/zenodo.17239356. ^18^ License: CC0 1.0 Universal.
