# Supplementary material for: Antioxidant Support for the Corneal Endothelium: Evidence from Vitamin C Supplementation in Hard Cataract Phacoemulsification
Source: F1000Res. 2026 Apr 24;14:1117. Originally published 2025 Oct 15. [Version 3] doi: 10.12688/f1000research.170846.3 (PMC13146457; doi:10.12688/f1000research.170846.3)
Supplement: Peer review history

# Reviewer report: Reviewer response for version 3

**DOI:** 10.5256/f1000research.197958.r480069

**Published:** 2026-05-15

**Authors:** Andrew Tirsi

**Affiliations:** 1 Manhattan Eye, Ear, and Throat Hospital, Northwell Health, New York, USA

This is not a research manuscript presenting results, and it does not look like a pilot study either.

 The initial submission was 6 months ago and one would expect some preliminary data presented and discussed. Instead, this is only a study protocol, describing future steps of a study, with missing results, discussion and conclusion sections.

 Please find some recommendations below:

 1-The background is short, The last sentence is confusing, because as it is now, it describes the protective effects of AA in patients with hard nuclear cataracts (HNC). There is no mention of phacoemulsification (P). Therefore, the rationale of this research is missed. Phacoemulsification in HNC is very specific and authors should add in this work why specifically this type of cataracts is investigated here. Is it because the nucleus is very hard, high ultrasound power is used, more turbulence created during the procedure. Maybe this should be described in details to explain why this study is conducted.

 2- Definitions should be added for HNC, and difficulties to make capsulorhesis when there is no red reflex, and other moments that make this procedure difficult and detrimental to the endothelial cells.

 3-Introduction is missing references after every significant statement, risks for plagiarism 

 4-The introduction is written in blocks, every paragraph not really linked to the previous one. not engaging the reader.

 The gap of knowledge is not mentioned, and therefore the purpose of this study in not clear.

 The last 3 lines in the introduction are not correct, because aqueous humor sampling can be done by aspiration (paracentesis) at the end of the surgery, and the use of experimental biosensors are used in research (ocucheck) that measures the electrical resistance of the aqueous humor for a quick quantitative analysis of vit c levels.

 5-How was 7 days of vit c supplementation decided?

 6-In the participants paragraph, please replace consume prescription by a better medical term

 7-Why vit c was used in this study? and what was the advantage of oral supplementation? how about topical? 

 8-will the side effects from the vit c supplementation monitored and registered?

 8- please explain in details the aqueous humor sampling, safety justification and possible side effects. How is the second sample collected after 3 minutes after the surgery. Aren't the authors concerned about the volume of the saline used? this is going to be a cofounding factor, or diluting factor for the MDA levels. How to correct for this? Will Elisa results be affected by this dilution factor?

 9- The manuscript initially was submitted in September 2025. I am sure authors could present some preliminary results to improve this manuscript in May 2026 (7-8 months)

Is the study design appropriate for the research question?

Partly

Is the rationale for, and objectives of, the study clearly described?

Partly

Are sufficient details of the methods provided to allow replication by others?

Partly

Are the datasets clearly presented in a useable and accessible format?

Not applicable

Reviewer Expertise:

cataract surgery, glaucoma, glaucoma surgery

I confirm that I have read this submission and believe that I have an appropriate level of expertise to confirm that it is of an acceptable scientific standard, however I have significant reservations, as outlined above.

# Reviewer report: Reviewer response for version 3

**DOI:** 10.5256/f1000research.197958.r478734

**Published:** 2026-05-05

**Authors:** Suraj Kumar Chaurasiya

**Affiliations:** 1 Department of Optometry and Vision Science, CL Gupta Eye Institute, Moradabad, Uttar Pradesh, India

Thank you for considering the suggestion and making changes as per the comment. No further comment to be made.

Is the study design appropriate for the research question?

Partly

Is the rationale for, and objectives of, the study clearly described?

Partly

Are sufficient details of the methods provided to allow replication by others?

Partly

Are the datasets clearly presented in a useable and accessible format?

Partly

Reviewer Expertise:

Contact Lens, Optometrist, Ocular disease, Optics, refractive surgery, Dry eyes

I confirm that I have read this submission and believe that I have an appropriate level of expertise to confirm that it is of an acceptable scientific standard.

# Reviewer report: Reviewer response for version 2

**DOI:** 10.5256/f1000research.196193.r464837

**Published:** 2026-03-12

**Authors:** Suraj Kumar Chaurasiya, Priyam Chaudhary

**Affiliations:** 1 Department of Optometry and Vision Science, CL Gupta Eye Institute, Moradabad, Uttar Pradesh, India; 2 CL Gupta Eye Institute, Moradabad, Uttar Pradesh, India

1. Knowledge Gap is Not Strongly Defined

- The manuscript states that no clinical study has investigated oral vitamin C supplementation in hard nuclear cataracts, but the **specific gap in the literature is not strongly emphasized**.

The introduction should clearly state:

- What is already known from previous antioxidant studies.
- What remains unknown regarding **systemic vitamin C supplementation and endothelial protection**.

2. The rationale does not adequately explain **why oral vitamin C supplementation is chosen instead of other antioxidant strategies**, such as:

- topical antioxidants
- Why **1500 mg/day** was chosen.
- intracameral antioxidants
- antioxidant-enriched irrigation solutions.
- Clarifying this would strengthen the translational relevance of the study.

3. The manuscript would benefit from a clear hypothesis, for example:

 Oral vitamin C supplementation increases intraocular antioxidant capacity and reduces oxidative stress–related endothelial damage during phacoemulsification.

 4. The study includes **120 participants (40 per group)**, but **no sample size calculation** is provided.

 Important details required:

- Primary outcome used for power calculation.
- Expected difference in **endothelial cell density (ECD) loss**.
- Assumed standard deviation.
- Alpha and power values.

5. Without this information, the adequacy of the study design cannot be evaluated.

 Although multiple outcomes are mentioned (ECD, CCT, visual acuity, inflammatory cells, oxidative stress markers), the **primary endpoint is unclear**.

 The manuscript should clearly specify:

 Primary outcome: e.g., **percentage endothelial cell loss at 6 weeks**

 Secondary outcomes: CCT, oxidative stress markers, visual outcomes, postoperative inflammation.

 6. This clarification is essential for RCT reporting.

 The manuscript states that the trial is **double-blinded**, but details are lacking.

 Required clarifications:

- Who generated the randomization sequence?
- Allocation concealment method (sealed envelopes, pharmacy randomization, etc.).
- Who is blinded?
  - patients
  - surgeons
  - outcome assessors
  - laboratory investigators.

7. Without this information, the risk of bias cannot be assessed.

 Endothelial cell loss during phacoemulsification strongly depends on intraoperative factors such as: cumulative dissipated energy (CDE), phaco time, ultrasound power, irrigation volume, viscoelastic type.

 8. The protocol states that identical machines and consumables will be used, but **intraoperative parameters should also be recorded and analyzed**.

 These should be included as covariates.

 9. The protocol involves **two aqueous humor samples during surgery**.

 Although small volumes (0.2 mL) are used, the manuscript should:

- provide a **safety justification**
- cite previous studies using similar sampling volumes
- explain whether this affects anterior chamber stability.

10. The statistical section needs refinement. Issues:

- Multiple group comparison methods are not specified.
- It is unclear whether **ANOVA or Kruskal-Wallis tests** will be used for the three-group comparison.
- Adjustment for **multiple comparisons** should be described.
- Multivariable analysis may be required to control for confounders such as:
  - age
  - nuclear grade
  - phaco energy.

11. "stress oxidative" Should be corrected to: **oxidative stress**

 12. Original: "Permanent endothelial decompensation usually observed 4–6 weeks postoperatively" Rewrite as: Permanent endothelial decompensation is typically observed 4–6 weeks postoperatively

 13. The first group description states: "receive oral vitamin C, tid" The dose must be explicitly written as: **500 mg vitamin C three times daily (1500 mg/day)**

 14. The manuscript does not clearly state: **Institutional Review Board approval number and **date of approval. These must be included.

 Include a **CONSORT flow diagram** for screening, randomization, follow-up, analysis.

 The protocol is interesting and potentially clinically impactful. However, clarification of the **study design, statistical plan, and methodological details** is necessary before publication.

Is the study design appropriate for the research question?

Partly

Is the rationale for, and objectives of, the study clearly described?

Partly

Are sufficient details of the methods provided to allow replication by others?

Partly

Are the datasets clearly presented in a useable and accessible format?

Partly

Reviewer Expertise:

Contact Lens, Optometrist, Ocular disease, Optics, refractive surgery, Dry eyes

We confirm that we have read this submission and believe that we have an appropriate level of expertise to confirm that it is of an acceptable scientific standard, however we have significant reservations, as outlined above.

# Response

**Published:** 2026-03-20

**Authors:** Syska Widyawati

**Affiliations:** Ophthalmology, University of Indonesia, Jakarta, DKI Jakarta, Indonesia

We sincerely thank the reviewer for the positive evaluation of our protocol and for the constructive comments. We appreciate the recognition of its potential clinical impact. In response, we have carefully revised the manuscript to clarify the study design, statistical approach, and key methodological details as suggested.

 1. We agree that the knowledge gap was not sufficiently highlighted in the original manuscript. The Introduction has been revised to better clarify the current evidence regarding antioxidant use in cataract surgery and to emphasize the remaining gap concerning systemic vitamin C supplementation and its potential protective effect on corneal endothelial cells, particularly in patients with hard nuclear cataracts. The revised paragraph now distinguishes what has been established in previous studies and what remains unclear, thereby strengthening the rationale for the present study.

 2. We agree that the rationale for selecting oral vitamin C supplementation should be more clearly explained. The Introduction has been revised to clarify the advantages of systemic vitamin C administration compared with other antioxidant strategies. In particular, oral vitamin C supplementation may provide a practical and widely accessible approach with a lower risk of adverse effects that may enhance systemic and intraocular antioxidants. 

 We have also clarified the rationale for selecting a dose of 1500 mg/day. Previous pharmacokinetic studies have shown that vitamin C supplementation at this dose can produce stable plasma concentrations and approach a steady-state level during short-term administration. This dosing strategy was selected to ensure adequate systemic antioxidant availability prior to surgery. This explanation has been added to the Introduction, and the dosing details are further described in the Intervention section.

 3. We agree that clearly stating the study hypothesis would improve the clarity of the manuscript. Accordingly, a hypothesis statement has now been added at the end of the Introduction to better define the expected effects of oral vitamin C supplementation on intraocular antioxidant capacity and oxidative stress–related endothelial damage during phacoemulsification.

 4. We agree that the sample size calculation should be clearly described. The Sample size calculation section has been revised to include details of the sample size estimation. The calculation was based on the primary outcome of postoperative corneal endothelial cell density (ECD) loss. The expected difference between groups and the assumed standard deviation were derived from previously published studies evaluating endothelial cell loss after phacoemulsification. A significance level (α) of 0.05 and a statistical power of 80% were used to determine the minimum sample size required for each group. The relevant details have now been added to the Sample size calculation section of the Methods.

 5. We agree that the primary and secondary outcomes should be more clearly defined to improve the clarity of the study design. The Aim section of the Methods has been revised to explicitly state the primary outcome and secondary outcomes of the study. The primary outcome will be defined as the mean change and percentage change in corneal endothelial cell density (ECD) following phacoemulsification. Secondary outcomes will include other clinical parameters such as central corneal thickness (CCT), visual acuity, postoperative anterior chamber inflammation, as well as the mean differences and correlations of oxidative stress biomarkers measured in blood and aqueous humor. These clarifications have now been added to the Aim section of the Methods.

 6. We agree that additional details regarding the randomization and blinding procedures are necessary to ensure transparency in reporting the randomized controlled trial. The Randomization, blinding and allocation concealment section of the Methods has been revised to clarify the process of randomization, allocation concealment, and blinding. Specifically, the randomization sequence will be generated using computer-based block randomization. Allocation concealment will be maintained using sequentially numbered, sealed, opaque envelopes that will be opened only after participant enrollment. Blinding will be maintained for operating surgeons, examiners, laboratory analysts, and study participants. These clarifications have now been added to the Randomization, blinding, and allocation concealment section of the Methods.

 7. We agree that intraoperative factors may influence corneal endothelial cell loss during phacoemulsification and should be clearly described to reduce the potential risk of bias. The Intervention section has been revised to clarify that the surgical procedures will be performed using standardized techniques and equipment to minimize variability in intraoperative parameters. All surgeries will be conducted by experienced cataract surgeons using the same phacoemulsification machine and standardized surgical settings, including ultrasound power,  irrigation parameters, and the same type of ophthalmic viscosurgical device (OVD). These measures will help minimize procedural variability and reduce the influence of intraoperative factors on endothelial outcomes.

 8. We agree that intraoperative factors may influence endothelial cell outcomes during phacoemulsification. To address this, the Methods section (post-intervention evaluation and statistical analysis section) has been revised to specify that intraoperative parameters, including effective phacoemulsification time (EPT) and total surgical duration, will be recorded for each procedure. These variables have now been added to  the statistical analysis as covariates to account for their potential influence on postoperative endothelial cell loss and other clinical outcomes.

 9. We agree that clarification regarding the safety of aqueous humor sampling is necessary. The Post-intervention section of the Methods has been revised to explain that the volume of aqueous humor collected (0.2 mL) is within the range commonly used in ophthalmic research and is considered safe when performed under controlled surgical conditions. Previous studies investigating biochemical markers in aqueous humor during cataract surgery have also used similar sampling volumes without reported adverse effects. In addition, aqueous humor sampling will be performed twice, and because the sampling will be performed through the surgical incision and the anterior chamber will subsequently be maintained with balanced salt solution during the procedure, this approach will not compromise anterior chamber stability. Relevant references describing similar aqueous humor sampling volumes will be added to the manuscript.

 10. The Statistical Analysis section has been revised to clarify the analytical approach used for comparisons among the three study groups. For normally distributed numerical variables, one-way analysis of variance (ANOVA) will be used, whereas the Kruskal–Wallis test will be applied for non-normally distributed data. When significant differences are detected, post-hoc pairwise comparisons with appropriate adjustment for multiple testing will be performed.

 In addition, multivariable analysis will be conducted to account for potential confounding variables that may influence oxidative stress status and postoperative outcomes. These variables include smoking status, occupational exposure, ultraviolet (UV) light exposure, and dietary intake of vitamin C. These factors will be included in the analysis to minimize potential confounding effects on the association between vitamin C supplementation and study outcomes.

 11. Thank you for pointing this out. The terminology has been corrected to “oxidative stress” throughout the manuscript.

 12. Thank you for the suggestion. The sentence has been revised accordingly to improve grammatical clarity.

 13. The dosage description has been revised to “500 mg vitamin C three times daily (1500 mg/day)” in the manuscript.

 14. The Institutional Review Board approval number and date of approval have now been added to the Ethics Statement section of the manuscript. In addition, a CONSORT flow diagram illustrating participant screening, randomization, follow-up, and analysis has been included to improve the clarity of the study design and reporting. The flow diagram have now been added to the Randomization, blinding, and allocation concealment section of the Methods

 We hope that these revisions have adequately addressed the reviewer’s concerns and have improved the clarity and rigor of the manuscript. We are grateful for the insightful feedback, which has significantly strengthened the quality of this work.

# Reviewer report: Reviewer response for version 2

**DOI:** 10.5256/f1000research.196193.r456960

**Published:** 2026-02-19

**Authors:** Rajesh Choudhary

**Affiliations:** 1 Shri Shankaracharya College of Pharmaceutical Sciences, Bhilai, Chhattisgarh, India

Authors addressed all my queries and suggestions.

Is the study design appropriate for the research question?

Yes

Is the rationale for, and objectives of, the study clearly described?

Yes

Are sufficient details of the methods provided to allow replication by others?

Partly

Are the datasets clearly presented in a useable and accessible format?

Partly

Reviewer Expertise:

preclinical studies on animal for anticataract drugs

I confirm that I have read this submission and believe that I have an appropriate level of expertise to confirm that it is of an acceptable scientific standard.

# Reviewer report: Reviewer response for version 1

**DOI:** 10.5256/f1000research.188354.r424633

**Published:** 2025-11-05

**Authors:** Rajesh Choudhary

**Affiliations:** 1 Shri Shankaracharya College of Pharmaceutical Sciences, Bhilai, Chhattisgarh, India

The study protocol was well designed. The study protocol lacks some details:

 1. Level of significance not to mention: Before the statistical analysis of the data, significance level must be defined like P < 0.05 or P < 0.01

 2. detailed procedure or citation not available for estimation of AA, MDA, and TCA. For reproducibility of the research, author must be clear defined the assay procedures, techniques, and methods used to measure the biochemical parameters.

 3. Oxidative stress markers  are measured in serum only, while Phacoemulsification produces free radicals on eyes. so explain any justification or try to evaluate oxidative stress markers in aqueous humor also.

Is the study design appropriate for the research question?

Yes

Is the rationale for, and objectives of, the study clearly described?

Yes

Are sufficient details of the methods provided to allow replication by others?

Partly

Are the datasets clearly presented in a useable and accessible format?

Partly

Reviewer Expertise:

preclinical studies on animal for anticataract drugs

I confirm that I have read this submission and believe that I have an appropriate level of expertise to confirm that it is of an acceptable scientific standard, however I have significant reservations, as outlined above.

# Response

**Published:** 2025-11-06

**Authors:** Syska Widyawati

**Affiliations:** Ophthalmology, University of Indonesia, Jakarta, DKI Jakarta, Indonesia

We sincerely thank the reviewer for the careful evaluation and valuable feedback on our study protocol. We appreciate these constructive suggestions, which have helped us improve the clarity and methodological rigor of our work.

**1. Level of significance:**

 We agree with the reviewer and will specify the significance level for statistical analysis as P < 0.05. This will be added in the section “Statistical Analysis.”

**2. Detailed procedure for estimation of AA, MDA, and TCA:**

 We appreciate this valuable suggestion. We will provide detailed information and appropriate citations for the methods used to estimate ascorbic acid (AA), malondialdehyde (MDA), and total antioxidant capacity (TCA). These details will be included in the revised version under “Laboratory Analysis.”

**3. Oxidative stress markers in serum versus aqueous humour:**

 We thank the reviewer for this valuable comment. In fact, oxidative stress markers were also measured in the aqueous humour samples. Sampling was performed twice, pre- and post-phacoemulsification. The first sample (0.2 mL of aqueous humour) was collected immediately after corneal incision using a 1 cc syringe (Terumo) with a 30 G cannula. The second sample (0.2 mL) was obtained after intraocular lens implantation and viscoelastic removal, approximately 3 minutes later, using the same technique. The pre-phacoemulsification aqueous humour samples were analysed for ascorbic acid (AA), total antioxidant capacity (TAC), and malondialdehyde (MDA), while the post-phacoemulsification samples were analysed for MDA levels only. These details will be clarified and incorporated in the *Post-intervention evaluation* section of the revised version.

 We sincerely thank the reviewer for the valuable and constructive feedback, which has strengthened the methodological transparency and scientific rationale of our research. We will prepare a revised version to address these points in detail.
